# The Use of Virtual Reality Facilitates Dialectical Behavior Therapy® “Observing Sounds and Visuals” Mindfulness Skills Training Exercises for a Latino Patient with Severe Burns: A Case Study

**DOI:** 10.3389/fpsyg.2017.01611

**Published:** 2017-09-25

**Authors:** Jocelyn Gomez, Hunter G. Hoffman, Steven L. Bistricky, Miriam Gonzalez, Laura Rosenberg, Mariana Sampaio, Azucena Garcia-Palacios, Maria V. Navarro-Haro, Wadee Alhalabi, Marta Rosenberg, Walter J. Meyer, Marsha M. Linehan

**Affiliations:** ^1^Department of Psychology, Shriners Hospitals for Children—Galveston Galveston, TX, United States; ^2^Clinical Health and Applied Sciences, University of Houston–Clear Lake Houston, TX, United States; ^3^Virtual Reality Research Center at the Human Photonics Lab, Mechanical Engineering Department, University of Washington Seattle, WA, United States; ^4^Department of Psychiatry and Behavioral Sciences, University of Texas Medical Branch Galveston, TX, United States; ^5^Psychology Department, Jaume I University Castellón de la Plana, Spain; ^6^Centro de Investigación Biomédica en Red-Fisiopatología de la Obesidad y Nutrición (CIBERobn) Madrid, Spain; ^7^Hospital General de Catalunya Barcelona, Spain; ^8^Virtual Reality Research Center, Computer Science Department, Effat University Jeddah, Saudi Arabia; ^9^Behavioral Research and Therapy Clinics, Department of Psychology, University of Washington Seattle, WA, United States

**Keywords:** virtual reality, burn patients, mindfulness, dialectical behavioral therapy, emotions

## Abstract

Sustaining a burn injury increases an individual's risk of developing psychological problems such as generalized anxiety, negative emotions, depression, acute stress disorder, or post-traumatic stress disorder. Despite the growing use of Dialectical Behavioral Therapy® (DBT®) by clinical psychologists, to date, there are no published studies using standard DBT® or DBT® skills learning for severe burn patients. The current study explored the feasibility and clinical potential of using Immersive Virtual Reality (VR) enhanced DBT® mindfulness skills training to reduce negative emotions and increase positive emotions of a patient with severe burn injuries. The participant was a hospitalized (in house) 21-year-old Spanish speaking Latino male patient being treated for a large (>35% TBSA) severe flame burn injury.

**Methods:** The patient looked into a pair of Oculus Rift DK2 virtual reality goggles to perceive the computer-generated virtual reality illusion of floating down a river, with rocks, boulders, trees, mountains, and clouds, while listening to DBT® mindfulness training audios during 4 VR sessions over a 1 month period. Study measures were administered before and after each VR session.

**Results:** As predicted, the patient reported increased positive emotions and decreased negative emotions. The patient also accepted the VR mindfulness treatment technique. He reported the sessions helped him become more comfortable with his emotions and he wanted to keep using mindfulness after returning home.

**Conclusions:** Dialectical Behavioral Therapy is an empirically validated treatment approach that has proved effective with non-burn patient populations for treating many of the psychological problems experienced by severe burn patients. The current case study explored for the first time, the use of immersive virtual reality enhanced DBT® mindfulness skills training with a burn patient. The patient reported reductions in negative emotions and increases in positive emotions, after VR DBT® mindfulness skills training. Immersive Virtual Reality is becoming widely available to mainstream consumers, and thus has the potential to make this treatment available to a much wider number of patient populations, including severe burn patients. Additional development, and controlled studies are needed.

## Introduction

Getting severely burned (e.g., in a house fire, a car accident, or scalding water) is a physically and medically traumatic experience. Recovering from a large severe burn injury involves intense medical treatments such as repeated painful wound cleaning sessions, skin graft surgeries, and physical therapy skin stretching exercises (Hoffman et al., 2011). Patients who have sustained a large severe burn are at increased risk of developing psychological disorders such as depression, anxiety, post-traumatic stress disorder, social anxiety, and apprehension about their changed appearance (Meyer et al., 2007, 2012; Stoddard et al., 2011; Rosenberg et al., 2015). Burn patients often report an increase in negative thoughts and emotions, and a decrease in positive thoughts/emotions (Meyer et al., 2012; Murphy et al., 2015). Patients may experience intense negative emotions such as guilt (e.g., survivor guilt) or shame when thinking about the burn injury. They also may have recurring flashbacks about the accident and this can cause a patient to feel sad, angry, or afraid (Meyer et al., 2007; Rosenberg et al., 2015). Further, patients with large severe burns are often hospitalized for weeks or months while they recover from their burn injury. This can be a very stressful experience and emotions often fluctuate throughout their treatment (Meyer et al., 2012). It is important for patients to learn effective coping skills when the patient is adjusting to the difficult changes associated with having a major burn injury. Although, current psychological interventions [e.g., Cognitive Behavioral Therapy (CBT)] can help burn patients adjust to psychological issues and stress, some new “simple to administer” psychological interventions that could further help patients learn adaptive coping skills would be welcomed.

Mindfulness is one intervention/coping skill that has been shown to improve mood and reduce stress. Mindfulness involves bringing ones attention into the present moment; bringing full awareness into present experiences on purpose and nonjudgmentally (Linehan, 2015). Dialectical Behavioral Therapy uses mindfulness to help patients overcome psychologically unhealthy thought processes and behaviors.

### Dialectical behavioral therapy (DBT®)

Standard DBT® treatment includes four main elements: individual psychotherapy sessions, phone coaching, skills training, and a therapist consultation team (to help the primary therapist conceptualize a patient). Certification to use this treatment requires extra training, beyond what is required to get licensed to practice psychotherapy. Although, a growing number of therapist are getting trained to use the full treatment, demand for DBT® far outstrips current clinical resources. Most patients who could benefit from standard treatment, never receive it. For example, there are no studies on PUBMED using DBT® with a burn patient.

During the past few years, non-standard “DBT® informed treatments” (Dimeff and Koerner, 2007) have been used (Linehan, 2015). Therapists who have not received the additional advanced training and supervision needed to become certified (a pre-requisite to using the full standard treatment), can still use DBT® skills learning modules adjunctively. For example, they can use traditional CBT instead of DBT® for the “one-on-one” therapy treatments, but then also add DBT® skills learning modules during group sessions.

DBT® skills training is a valuable element of Standard DBT®. In one study by Linehan et al. (2015), Borderline Personality Disorder patients received DBT® with and without the skills training component. Adding the skills training significantly improved psychotherapy efficacy/clinical outcomes for suicidal patients (predominantly female) diagnosed with borderline personality disorder (Linehan et al., 2015). In other studies, DBT® skills training, stand-alone, has been shown to increase attention and reduce impulsivity (Soler et al., 2012, see also Valentine et al., 2015; Kramer, 2017).

### Dialectical behavioral therapy skills training

DBT® skills training covers four main categories: mindfulness, emotion regulation, effectiveness in relationships, and distress tolerance (Linehan, 1993, 2015). During skills training, patients are taught methods to help them deal more effectively with their own intense emotions (e.g., anger outbursts). Patients learn skills to help improve their relationships with other people. Patients learn skills to reduce self-destructive behaviors. DBT® mindfulness skills training is the most important skills module. Patients learn mindfulness skills first in order to help set the stage for learning other DBT® skills. Patients start by directing their attention toward simple perceptual stimuli (e.g., sights and sounds). Patients learn to accept these experiences without judgment (e.g., without self-criticism). They later learn how to experience emotions, thoughts, and sensations while still being able to focus their attention on an activity. The eventual goal is for patients to use mindfulness and acceptance skills in daily life, so they are encouraged to develop these skills by incorporating mindfulness practice into their daily routines. For example, a patient can notice feelings of anxiety, and can still participate effectively in a social activity.

Early in their mindfulness skills training, patients often find it challenging to focus their attention, as directed by the therapist. Many patients have trouble building the basic foundation for attentional focus and flexibility in the presence of other competing stimuli (e.g., unhealthy intrusive thoughts). Patients can become discouraged by their difficulty concentrating, and decrease practice, stunting how much they benefit from mindfulness. Although, starting with basic mindfulness exercises and working toward more challenging exercises over time (i.e., scaffolding mastery) can address part of this problem, other facilitators of patient engagement and mastery with mindfulness exercises, such as increasing attentional focus using immersive virtual reality, might provide significant benefits (Navarro-Haro et al., 2016).

### Virtual reality

Immersive virtual reality involves wearing/looking into VR goggles as a window into a 3D simulation. Immersive VR is designed to make the patient feel “present” in the virtual world, as if the computer generated world is a place they are visiting (Slater and Wilbur, 1997).

Previous studies have explored the use of “nature scene” themed immersive virtual reality to help severe burn patients (see Hoffman et al., 2011 for a review). Patients with severe burns had the illusion of going inside an icy 3d virtual reality canyon nature scene named SnowWorld (http://www.vrpain.com). Patients who went into SnowWorld during painful wound care sessions reported significant reductions in negative emotions (pain unpleasantness), and reported significant increases in positive emotions (Maani et al., 2011). Recently, a laboratory study on healthy non-patients by another group of researchers showed that in non-patients, visiting an immersive nature scene in virtual reality significantly reduced negative emotions, compared to “being inside a building, in virtual reality” (Anderson et al., 2017), so “being outdoors in nature” when in virtual reality, significantly reduced negative emotions.

The essence of immersive virtual reality is the participant's illusion of visiting a different (computer generated) world. “Being there” in this rich, novel VR environment engages a significant portion of the participant's attentional resources, such that other competing stimuli are rendered comparably less potent. Immersive VR blocks patients' view of the real world (Hoffman et al., 2000; Hoffman, 2004), and may help patients focus their attention on the mindfulness skills exercise in VR (Navarro-Haro et al., 2016). Virtual Reality DBT® mindfulness skills learning is a simple new psychological technique that combines mindfulness with immersive virtual reality. The patient goes into a computer generated virtual reality world, and practices mindfulness in virtual reality.

Despite their growing use by clinical psychologists, to date, there are no published studies using standard DBT® or DBT® skills training to help train psychological coping skills (e.g., mindfulness) to severe burn patients. The current study explored if it is feasible for burn patients to practice mindfulness while in virtual reality and whether VR DBT® could help reduce negative emotions and increase positive emotions in a patient with large severe burn injuries. In the current study, the burn patient was reporting difficulty coping with his thoughts and emotions, and had difficulty concentrating. The VR DBT® treatment focused on reducing negative emotions often experienced by burn patients. Most basic emotions are negative emotions, (Ekman, 1992, 2016). For these reasons, we measured five negative emotions and only one positive emotion. In the current study, we hypothesized that: (1) The patient would experience a decrease in negative emotions and an increase in positive emotions after each VR DBT® mindfulness skills training session. (2) We further predicted that the patient would accept VR for learning DBT® mindfulness skills. Given the study's focus on feasibility and the fact that the patient could only complete a small number of VR sessions, significant long term changes in psychological symptoms or mindfulness skills were not anticipated or measured. However, we assessed post-traumatic stress symptoms to characterize this particular patient (who had experienced recent traumatic events).

## Materials and methods

The patient was fully informed of what the treatment would entail, before consenting, and he signed an informed consent form approved by the University of Texas Medical Branch IRB. The patient consented to allow the de-identified results of this study to be published in scientific journals. The participant did not receive compensation for participation in this study.

### Overview

The DBT® mindfulness training focused on two “Observing” skills, both designed to help patients learn to control where they directed their attention. In “observing sounds,” the patient practiced focusing his attention on sounds he heard in virtual reality, and practiced bringing his attention back to the sounds whenever his attention wandered. In “observing visuals,” the patient was invited to pay attention to the various visual stimuli in the virtual reality environment (Linehan, 2015).

### Participant

The subject was an unmarried single young adult Latino male hospitalized and receiving treatment for a severe skin burn covering over 1/3rd of his total body surface area after being severely injured during a fire. He had small first/second degree burns on his face, and second/third degree burns on several areas of his body. The patient did not receive standard DBT (e.g., he received CBT instead of standard DBT® during one-on-one therapy sessions, separate from the current study). He had never used mindfulness before, and had never used VR technology prior to the study. The inpatient had been in the hospital burn center for several months, recovering from his severe burn injury. He was seeing a licensed clinical psychologist for CBT, once a week, and was taking fluoxetine to help manage psychological symptoms related to his burns. During his CBT sessions, the patient reported being worried about returning home and as a result he was experiencing anxiety symptoms. Furthermore, he reported that anxiety and intrusive, ruminative thoughts impeded his concentration during the day. For example, his burn injury left scars on his body and he was concerned about the comments people might make about his appearance. The patient was also at times overwhelmed after his CBT psychotherapy sessions. Thus, the treating licensed clinical psychologist thought the patient might benefit from VR DBT® mindfulness skills training. The patient started this particular intervention during the third month of individual CBT (with no standard DBT®). He received VR DBT® mindfulness skills training modules once a week for 4 weeks (four VR sessions total).

### Therapist

The first author of this paper (JG) treated the patient using the VR DBT® mindfulness training. At the time this case study was conducted, JG was working on her clinical internship at the burn hospital, toward her Master's degree in clinical psychology. A licensed clinical psychologist supervised the therapist (JG) during the VR DBT® mindfulness skills training sessions. The therapist (JG) treated the patient as part of the multidisciplinary treatment team, and attended weekly supervision meetings with her clinical supervisor.

### Design and study procedures

The current case study used a within-subject design. The primary measurements were administered before and after each VR DBT® mindfulness skills training session. VR DBT® mindfulness skills training was incorporated as an adjunct to individual CBT (at the end of session) for 4 sessions.

**Figure 1:**
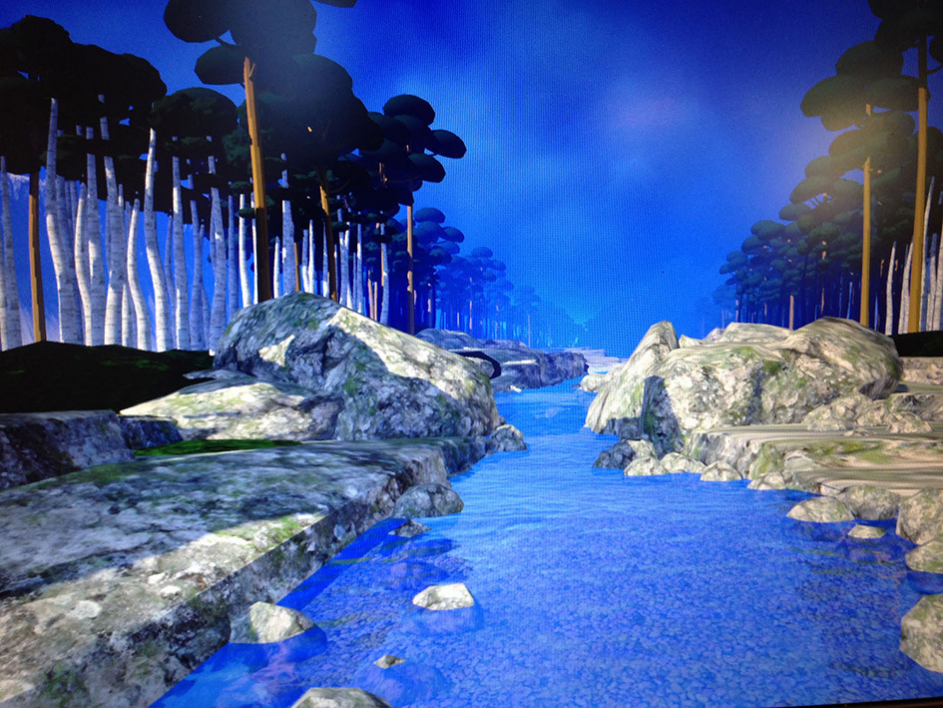
The patient had the illusion of floating down an animated river in immersive virtual reality, with rocks, boulders, trees, mountains, and clouds, while listening to DBT® mindfulness training audios (http://behavioraltech.org). The patient was asked to “be here now,” to notice the water, the rocks in the water, the boulders on the sides of the river, the trees, the mountains in the distance, the light, and the clouds. “If your mind wanders away, that's ok, just bring your thoughts back to the present moment.” Image by www.bigenvironments.com, copyright Hunter Hoffman, www.vrpain.com.

While looking into the head mounted/head tracked Oculus Rift DK2 VR goggles (with 100 degrees field of view and 960 × 1,080 pixels per eye, 75 Hz), the patient listened to mindfulness training instructions followed by sounds of birds and chirping, and water sounds, in an immersive computer simulation of floating down a 3-D computer-generated river during the VR DBT® mindfulness intervention (see Figure 1; VR software/visuals copyrighted by BigEnvironments.com, see also http://www.vrpain.com).

While looking into the virtual reality goggles, the patient was able to look around the virtual world. Near the beginning of the VR session, the patient stayed in one place near at the top of the virtual river, as he received verbal mindfulness skills training exercise instructions from his therapist. Then, the patient began descending slowly down the river while listening to audio instructions directing the patient how to practice mindfulness.

### Observing sound: (10 min total)

The therapist (JG) translated Dr. Linehan's “From Suffering to Freedom through Acceptance®” (Linehan, 2002) recording into Spanish, and read the script to the patient while the patient was in the virtual reality world. The therapist briefly described what mindfulness is for about 3.5 min. She explained how people often confuse mindfulness with meditation or relaxation and how people often expect to feel better after a mindfulness exercise. While in VR, the instructions helped train the patient how to “live in the present moment.” The patient was asked to “be here now.” “If your mind wanders away, that's ok, just bring your thoughts back to the present moment.” While in VR, the patient was instructed to focus his attention on sounds, the sounds of the water, and birds chirping, sounds he heard as he floated down the computer animated river in virtual reality. The patient was told to bring his attention back to noticing the sounds, whenever his mind wandered. The goal of mindfulness was to just notice and not necessarily to feel better. After hovering at the top of the river during the introduction, the patient began descending down the computer animated river in virtual reality while listening to water only or birds only sounds (via the VR computer speakers). The therapist guided the patient to bring his attention back to the sounds whenever his mind wandered off.

### Observing visuals: (10 min total)

Dr. Linehan's guided “observing visuals” audio was also adapted to Spanish by the therapist. In the beginning of the “observing visuals” VR DBT mindfulness sessions, the therapist briefly explained, for 1 min, the instructions for this exercise. The therapist guided the patient to observe what he was seeing in the VR goggles. The patient was asked to “be here now,” to notice the water, the rocks in the water, the boulders on the sides of the river, the trees, the mountains in the distance, the light, and the clouds. “If your mind wanders away, that's ok, just bring your thoughts back to the present moment.” The objective of this exercise was to only “observe the objects along the way” and to practice bringing their attention back to the objects and to the present moment, if the patient's mind wandered. Finally, the hospitalized burn patient was encouraged to practice mindfulness outside of session and to keep a record of how often he practiced mindfulness.

## Assessments

An adaptation of the DBT® diary cards (Linehan, 1993) was used to measure the intensity of the patient's primary emotions (fear, sadness, disgust, anger, guilt, shame, and joy) on a scale of 0–100 before and after each VR DBT® mindfulness session. The burn injured patient was encouraged to write any comments about the experience before and after each session. The PTSD CheckList-Civilian version (PCL-C) was administered at the start and at the conclusion of the intervention to examine post-traumatic stress symptoms. The PCL-C has good internal consistency and it is a common measure used to assess for PTSD (Spoont et al., 2013). A Graphic Rating Scale (GRS) of 0–10 (e.g., none of the time to all the time) was administered after each session to measure DBT® mindfulness acceptance. This scale measured how likely the patient would be to utilize mindfulness outside of session after his practice with VR DBT® mindfulness.

## Results

### Subject characteristics

The patient's PCL total scores prior to and following his completion of the four VR DBT® sessions (pre: 26; post: 21) suggest that the patient did not meet full criteria for post-traumatic stress disorder at either time point. But there was a small reduction in PTSD symptoms in the predicted direction, over the 1-month period.

### Acceptability and affect change

The patient's comments (translated into English, see Table 1) suggested that VR DBT® mindfulness was well accepted. The patient reported that this VR DBT® mindfulness intervention improved his mood and he believes this could help other patients. The patient also reported practicing mindfulness twice, outside of therapy.

**Table 1:** Detailed information about each VR DBT® Session.

**Session number**	**Stimuli**	**Comments about the experience**	**How likely are you to use this skill outside of session? On a scale of 0–10**	**Was DBT® mindfulness skills used outside of session?**
1	Patient visually observed the VR world (e.g., water, trees, boulders) for 10 min while being guided to bring attention back to the present moment if his mind wanders	This treatment program is an experience that changes our emotional state. It would be beneficial for patients who have suffered a trauma	8	No
2	Patient listened to water sounds while in the VR world and was guided to bring attention back to the sound if his mind wandered	Was initially upset because I learned I will be staying in the hospital longer than expected. I really enjoyed the water sound instead of only observing the visuals	8	Yes
3	Patient listened to bird sounds while in the VR world and was guided to bring attention back to the sound if his mind wandered	No comment	8	Yes
4	Patient visually observed the VR world while listening to bird sounds for 10 min and was guided to bring attention back to the sound if his mind wandered	This program is beneficial because it helps change our self-concept and our thoughts	8	No

**Figure 2:**
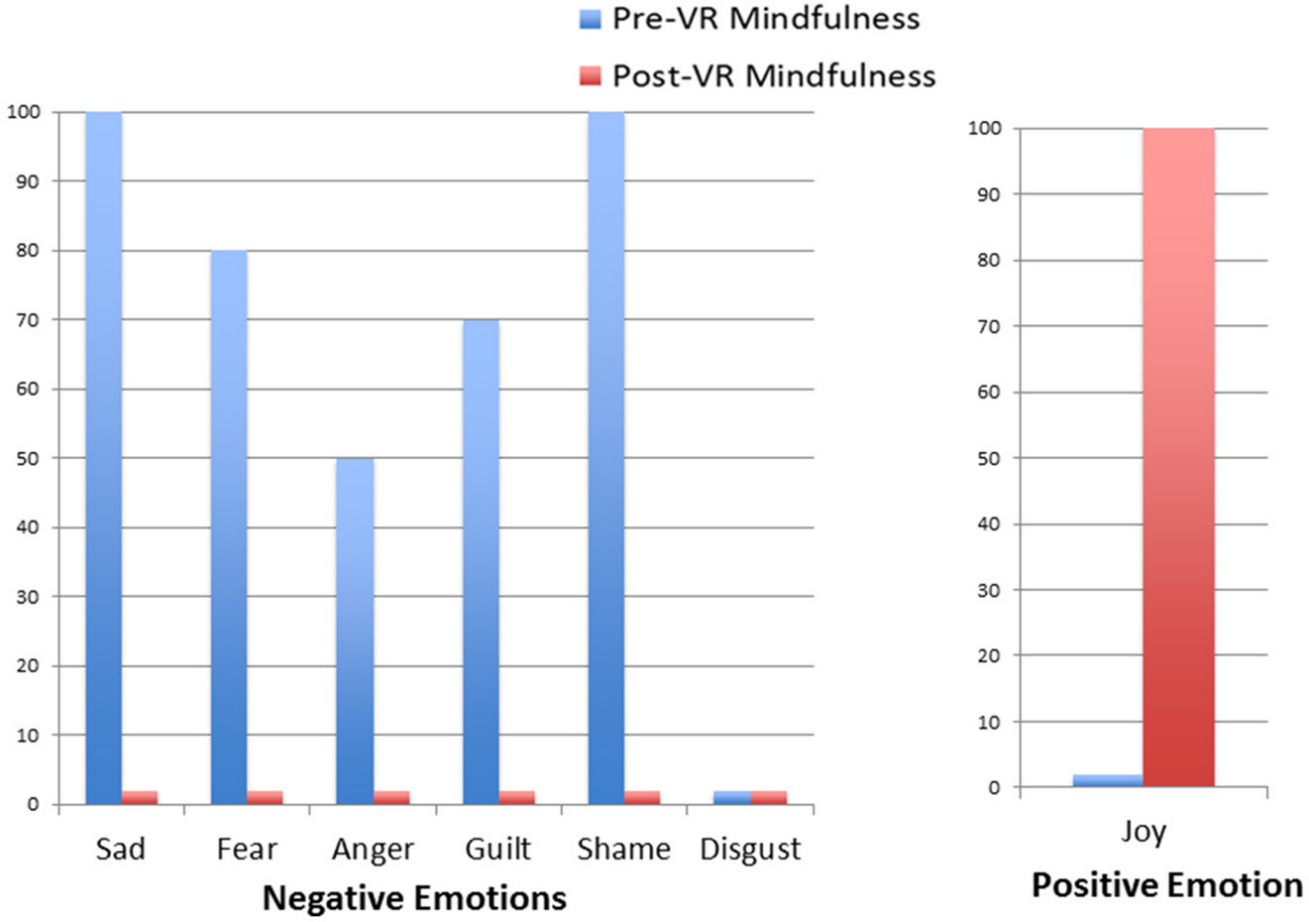
Emotions before and after VR DBT mindfulness session: Day 1–observing visuals.

**Figure 3:**
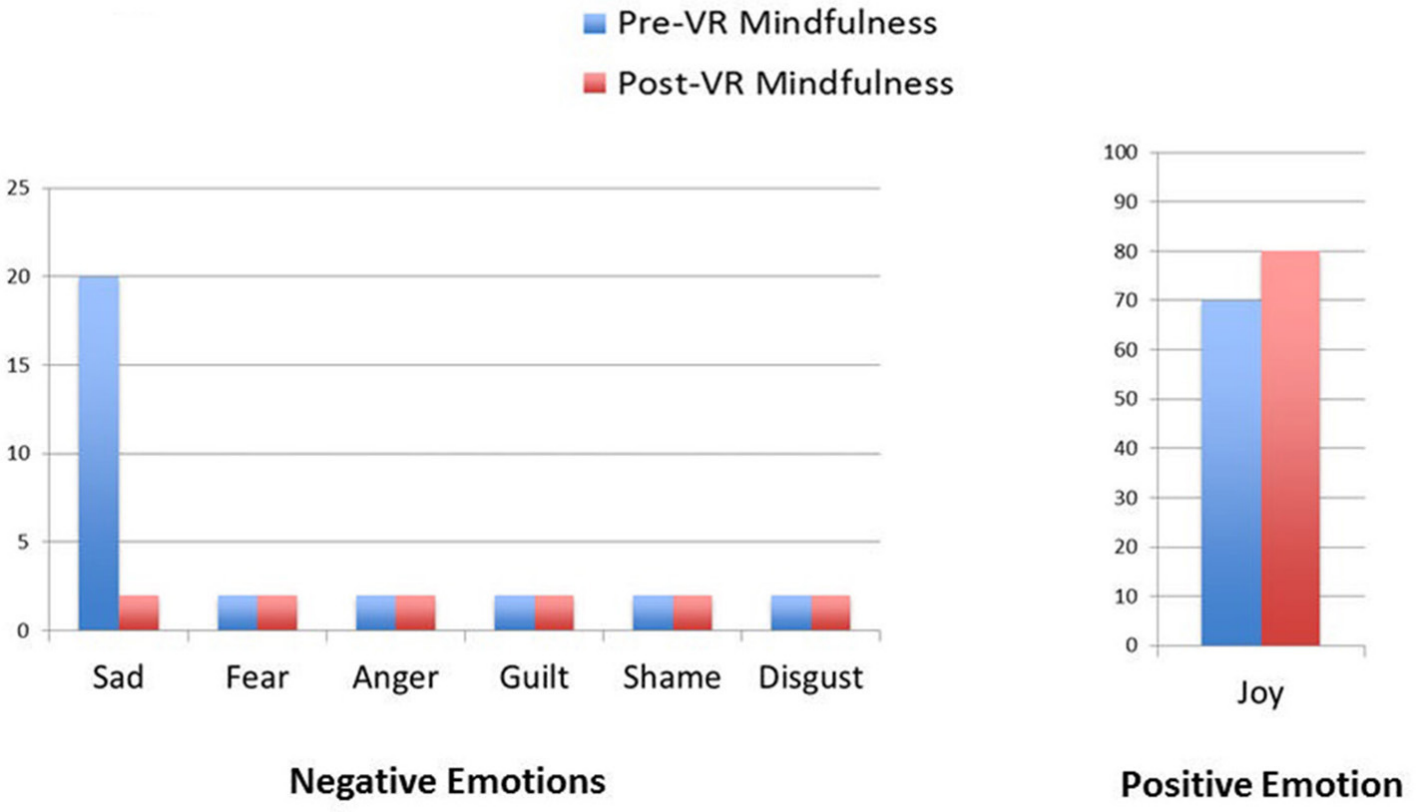
Emotions before and after VR DBT Mindfulness session: Day 2–observing water sounds.

**Figure 4:**
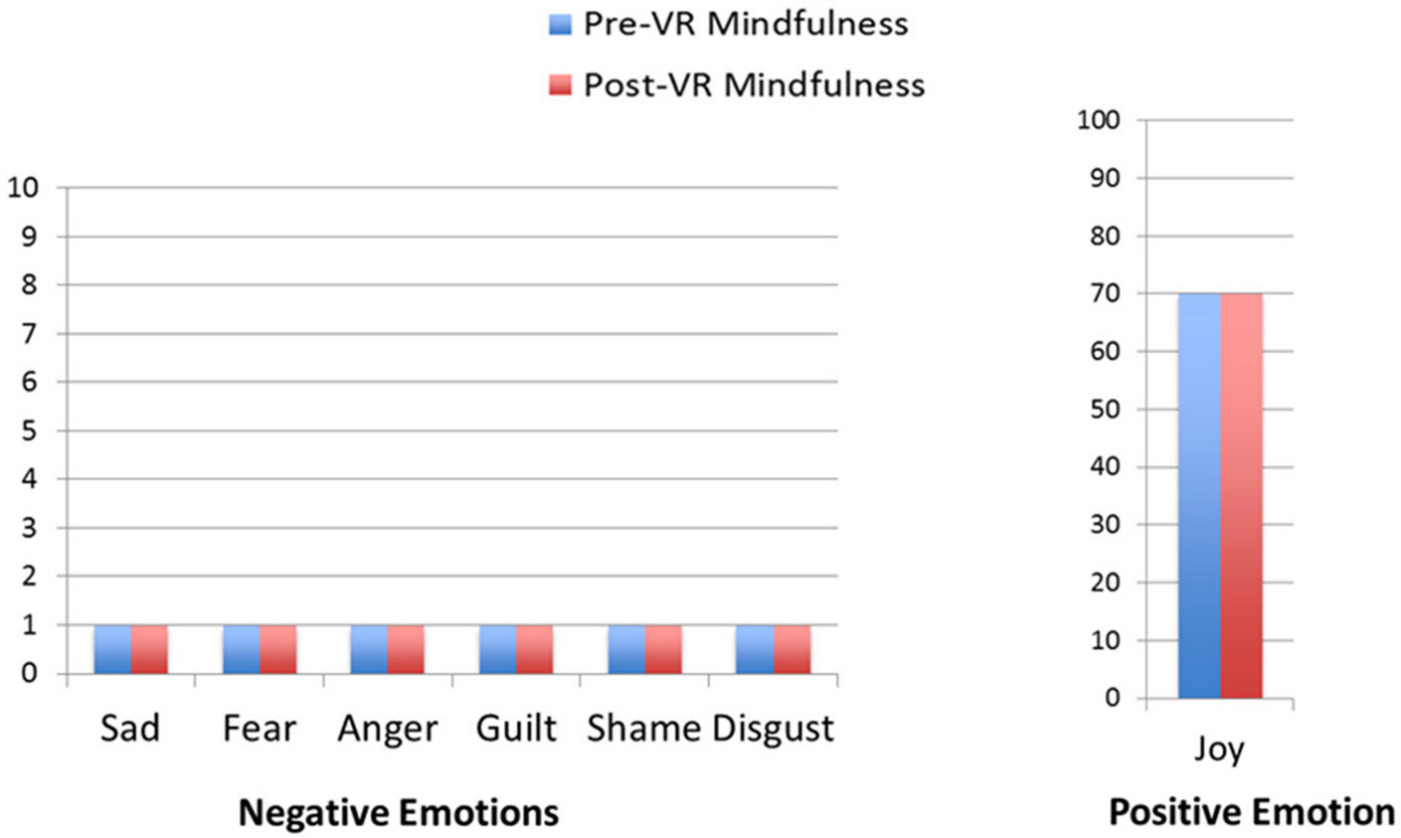
Emotions before and after VR DBT mindfulness session: Day 3–observing bird sounds.

**Figure 5:**
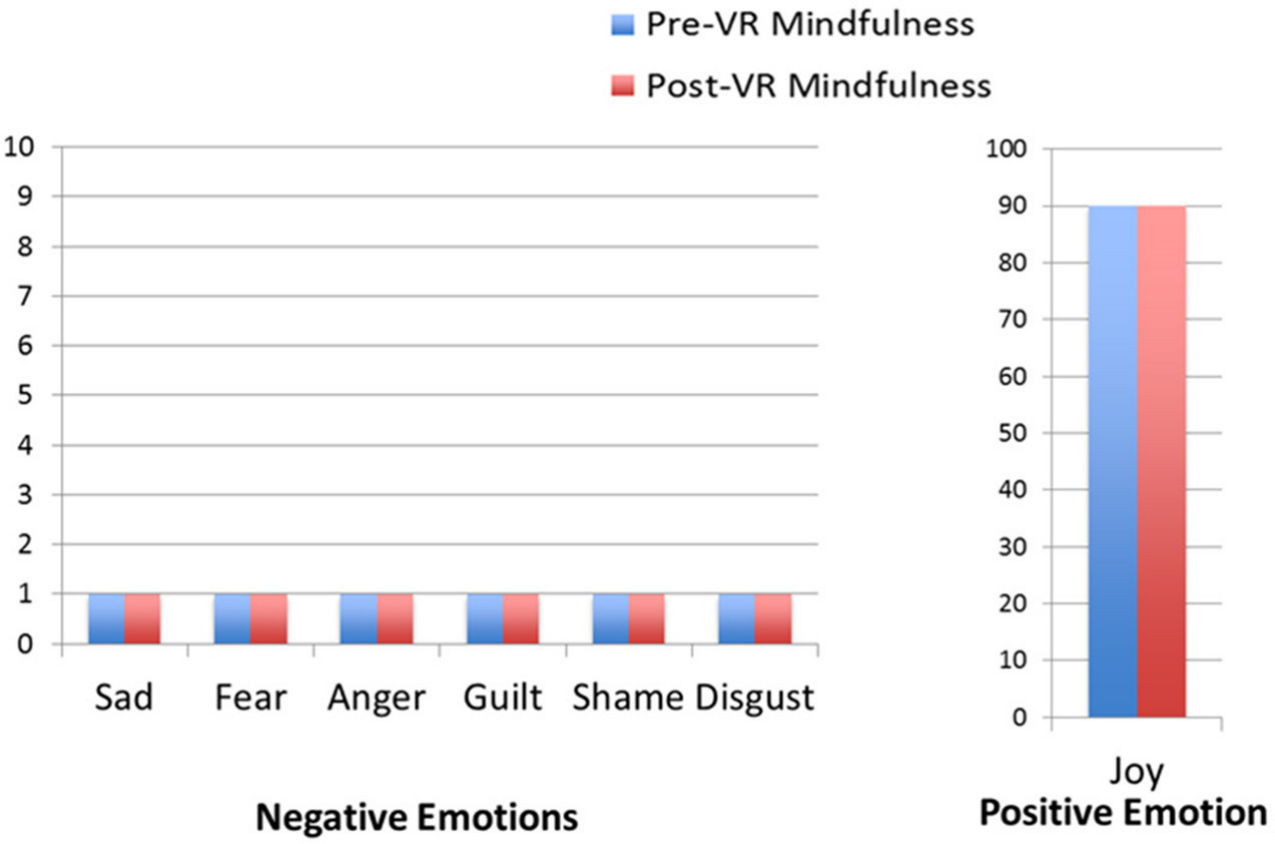
Emotions before and after VR DBT Mindfulness session: Day 4–observing visuals and sound.

Results regarding the patient's self-ratings of primary emotion intensity are shown in Figures 2–5. As predicted, the VR DBT® mindfulness exercises decreased negative emotions and increased positive emotions. The reduction in negative emotions was most pronounced on Day 1, (see Figure 2), but he also showed the predicted pattern during Day 2 (see Figure 3). On Days 3 and 4, (Figures 4, 5), there was a floor effect on negative emotions and a ceiling effect on positive emotions. Both before and after the VR DBT® mindfulness exercise, negative emotions were near zero, and positive emotions were very high. In other words, on Study Days 3 and 4, the patient was already doing so well that there was little or no room for measurable improvement from VR DBT® mindfulness training.

## Discussion

Based on the authors' review of the literature on PubMed, the current case study constitutes the first study to use DBT® mindfulness skills training with a burn patient (i.e., having the burn patient immersed in a computer generated virtual reality world during DBT® mindfulness skills training). In the current study, on the days he had negative emotions, a patient with a severe burn injury showed improvement in emotional state after VR DBT® mindfulness skills training. The patient accepted and reported benefitting from the treatment.

Although, promising, the current study has several limitations. Because it is not possible to make broad scientific conclusions based on the results of a single patient (Campbell and Stanley, 1963), case studies must be followed up with controlled studies. Another possible limitation is that the patient was receiving a broader treatment program, including CBT and a prescription medication. Fortunately, in the current study, our use of a within-subjects design measuring emotions before and after each treatment is designed to measure and isolate the effects of the VR DBT treatment.

The present study does not measure long-term benefits but is rather a preliminary study to explore whether burn patients would use virtual reality DBT mindfulness skills training, and whether the treatment reduced the patient's negative emotions and increased their positive emotions. Although, patients can find immediate short-term benefits with individual mindfulness exercises, significant long-term skill development would require considerable practice. For example, Mindfulness-Based Stress Reduction has been found to be effective in increasing change in mindfulness skills after an 8-week program (Baer et al., 2012). With practice, their ability to focus attention increases, and they can progress to directing their attention to their own thoughts, emotions, and sensations (Linehan, 2015). In the future, it is hoped that after training with VR DBT mindfulness skills training, eventually the burn patient can practice mindfulness and other valuable psychological skills, during their everyday lives, even when they don't have access to virtual reality. The results of the current feasibility study indicate VR DBT mindfulness skills training has potential, and there were no contraindications. In light of these encouraging outcomes (e.g., reduced negative emotions), in the next phase of this research program, we will begin to assess clinical outcome, using standardized long-term outcome measures.

Despite this study's limitations, the current findings provide encouraging initial support for the feasibility, acceptability, and clinical potential of using DBT® mindfulness skills training for a patient with a severe burn injury. Study findings are consistent with the rationale that Virtual Reality might increase the acceptability of DBT® mindfulness skills learning (Garcia-Palacios et al., 2001, 2007). In addition, computerization of DBT® skills modules has the potential to increase dissemination (Navarro-Haro et al., 2016). Whether VR DBT® mindfulness skills training has long term benefits for burn patients such as increased positive emotions, reduced negative emotions, reduced anxiety, reduced PTSD symptoms, reduced depression, and/or increased feelings of well-being are important topics for future research.

In addition to the need for additional research on “DBT® informed” treatments, future research is also needed on the clinical effectiveness of the full standard DBT® treatment for helping patients with severe psychological problems. The full “standard” treatment requires more resources, but could be especially effective for burn patients with psychological problems. There is a growing literature showing the effectiveness of DBT® for treating a number of psychological problems commonly faced by severe burn patients (e.g., anxiety, depression, chronic pain, PTSD, body image concerns), but to date, none of these studies have included severe burn patients. Additional research is needed to determine whether these new treatment options (DBT® and/or VR DBT® skills training) are effective for reducing long term burn-related psychological problems often suffered by burn patients during recovery and after they physically heal from their burn injury.

## Author contributions

All authors listed have made a substantial, direct and intellectual contribution to the work, and approved it for publication.

### Conflict of interest statement

ML, ABPP is the treatment developer of Dialectical Behavior Therapy (DBT). She receives licensing fees for Behavioral Tech's use of her training materials. The other authors declare that the research was conducted in the absence of any commercial or financial relationships that could be construed as a potential conflict of interest.
